# Prognostic Value of Circulating Cell-Free DNA Concentration and Neutrophil-to-Lymphocyte Ratio in Patients with Pancreatic Ductal Adenocarcinoma: A Prospective Cohort Study

**DOI:** 10.3390/ijms25052854

**Published:** 2024-03-01

**Authors:** Bianca Varzaru, Razvan Andrei Iacob, Stefania Bunduc, Ioana Manea, Andrei Sorop, Andreea Spiridon, Raluca Chelaru, Adina Croitoru, Mihaela Topala, Gabriel Becheanu, Mona Dumbrava, Simona Dima, Irinel Popescu, Cristian Gheorghe

**Affiliations:** 1 Faculty of Medicine, Carol Davila University of Medicine and Pharmacy, 020021 Bucharest, Romania; bianca.stoica@drd.umfcd.ro (B.V.); stefania.bunduc@drd.umfcd.ro (S.B.); elena-mihaela.topala@drd.umfcd.ro (M.T.); gabriel.becheanu@umfcd.ro (G.B.); drcgheorghe@gmail.com (C.G.); 2 Center of Excellence in Translational Medicine, Fundeni Clinical Institute, 022328 Bucharest, Romaniadmona98@yahoo.com (M.D.); irinel.popescu220@gmail.com (I.P.); 3 Digestive Diseases and Liver Transplantation Center, Fundeni Clinical Institute, 022238 Bucharest, Romania

**Keywords:** cell-free DNA, neutrophil-to-lymphocyte ratio, pancreatic ductal adenocarcinoma, overall survival

## Abstract

Circulating cell-free DNA (ccfDNA) quantity correlates with the clinical characteristics and prognosis of various cancer types. We investigated whether ccfDNA levels and the neutrophil-to-lymphocyte ratio (NLR) have prognostic value in patients with pancreatic ductal adenocarcinoma (PDAC). Peripheral blood was collected from 82 patients with PDAC prior to any diagnostic procedure or the administration of chemotherapy. Plasma DNA was isolated, and ccfDNA concentration and NLR were determined. We found that ccfDNA levels were correlated with age and tumor burden. Moreover, higher values of NLR (≥3.31) were linked with worse overall survival (OS) (4 vs. 10 months; log rank *p* = 0.011), and an elevated ccfDNA concentration (≥25.79 ng/mL) was strongly associated with shorter OS (4 vs. 8 months; log rank *p* = 0.009). According to the results of the multivariable Cox regression analysis, the baseline concentration of ccfDNA was an independent prognostic factor for OS (HR 0.45, 95% CI 0.21–0.97, *p* = 0.041). Furthermore, the combination of ccfDNA levels with NLR greatly enhanced the prognostic accuracy of PDAC patients. Our study demonstrates that ccfDNA concentration and NLR are independent predictors of survival in PDAC. Subsequent studies should validate this combination as a prognostic indicator in PDAC patients and assess its utility for guiding therapeutic decisions.

## 1. Introduction

Pancreatic ductal adenocarcinoma (PDAC) is a tumor with a dismal prognosis due to late-stage diagnosis and early metastasis, with an overall 5-year survival rate of less than 9% [1,2,3]. Due to their aggressive behavior, significant inter- and intra-cellular heterogeneity, and the abundance of desmoplastic microenvironments, PDAC is rather resistant to standard therapy, including chemo- and radio-therapy, as well as to targeted agents and immunotherapies [4,5].

Carbohydrate antigen 19-9 (CA19-9) is a common serum protein biomarker used to track treatment responses in PDAC; however, its use in predicting disease outcomes and treatment responses in advanced and metastatic cancer is unclear due to its weak correlation to imaging data for subsequent evaluation of responses [6].

There is evidence that inflammation plays a critical role in the development and progression of pancreatic cancer [7]. Inflammation has an impact on every step of carcinogenesis, including early growth, tumor promotion, and metastatic dissemination [8]. In numerous solid tumors, including pancreatic cancer, NLR has been suggested as a marker of the systemic inflammatory response [9,10].

Blood-based liquid biopsy biomarkers that are non-invasive and repeatable have been assessed as diagnostic and prognostic indicators [11]. Plasma ccfDNA is a minimally invasive biomarker that originates from cell lysis, apoptosis, necrosis, and the active release of DNA fragments into blood stream during tumorigenesis [12,13]. Tumor masses exhibit significantly higher levels of apoptosis due to the proliferation of cancer cells and rapid cell turnover. As a result, cellular debris that macrophages would typically phagocytose cannot be entirely eliminated; rather they accumulate, and are discharged in the blood circulation [14,15].

The release of ccfDNA differs between cancer types, and it is well documented that it is associated with advanced stage, tumor burden, and a high number of metastases [16]. Droplet digital PCR (ddPCR) or next-generation sequencing (NGS) are widely used techniques for prognostic biomarkers profiling in liquid biopsy, whereas the fluorometric assessment of the ccfDNA concentration provides a simple, easy-to-use, and accessible approach [17].

The purpose of this study was to investigate the correlations between plasma ccfDNA quantity and clinical and tumor features, as well as the prognostic value of plasma ccfDNA concentration and NLR in PDAC patients.

## 2. Results

### 2.1. Patient Characteristics

Clinicopathological data and baseline patient characteristics are shown in Table 1. The median age was 67 years, with an IQR of 62–70, and the male-to-female ratio was 1.21:1. The median tumor size was 40 mm (IQR, 32.75–50). The primary tumors were found in the pancreatic head or uncinate process in 51 patients (62.2%), whereas pancreatic body or tail tumors were found in 31 patients (37.8%). Venous and arterial invasion were observed in a significant proportion (43.9% of cases and 68.29%, respectively). In total, 9 of the 82 patients were in stage I (1.2% IA and 9.8% IB), 10 patients were in stage II (2.4% IIA and 9.8% IIB), 21 patients were in stage III (25.6%), and 42 patients were in stage IV (51.2%). Based on the outcomes of the lab tests, the normal value of CA 19-9 in our study was established as 39 U/mL.

Among the patients who were included, 76 patients (83.8%) had an ECOG-PS of 0/1, and 6 patients (7.3%) had an ECOG-PS of 2/3. Oligo-metastatic cancer affected 20.7% of the 47 patients initially diagnosed with metastatic disease.

### 2.2. Measurement of ccfDNA Concentration and Correlations with Clinical and Tumor Characteristics

The median plasma volume used in the isolation step was 8 mL (IQR, 7–10). The median ccfDNA concentration was 10.3 ng/mL (IQR, 5.1–21.46), ranging from a minimum of 1.15 ng/mL to a maximum of 78.33 ng/mL.

**Figure 1:**
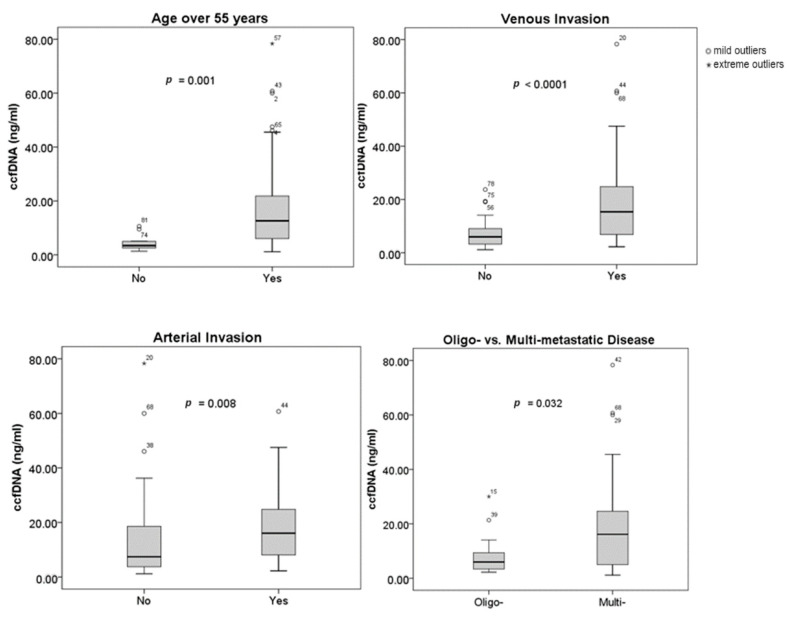
Correlation between ccfDNA concentration and clinical/tumor characteristics.

Figure 1 illustrates the relationship between the concentration of ccfDNA and clinical and tumor features. Age, vascular invasion, and the number of metastases at the time of diagnosis have all been found to be correlated with plasma ccfDNA levels. Ages over 55 were linked to higher ccfDNA concentrations (*p* = 0.001). Additionally, statistical correlations between greater ccfDNA levels and venous (*p* = 0.0001) and arterial invasion (*p* = 0.008), respectively, were found. While oligo-metastatic PDAC was associated with lower levels of ccfDNA, multi-metastatic disease was linked with higher ccfDNA concentrations (*p* = 0.032). Tumor size (*p* = 0.385), tumor location (*p* = 0.43), baseline CA 19-9 (*p* = 0.381), or NLR (*p* = 0.619) did not have a statistically significant correlation to the ccfDNA concentration. Neither diabetes mellitus (*p* = 0.926) nor ECOG-PS (*p* = 0.51) had an impact on the levels of ccfDNA.

### 2.3. Survival Analysis

Patients were followed for a median of 7 months (ranging from 1 to 47 months). The Cox regression model was used to assess the relationship between patients’ or tumor’ characteristics and overall survival. Both univariable and multivariable Cox regression analyses were performed to estimate the prognostic role of ccfDNA levels and NLR on survival, relative to other clinicopatological parameters. This analysis took into account factors like age, gender, presence of diabetes, tumor location, tumor size, tumor stage, metastatic status, baseline CA 19-9 levels, and NLR (dichotomized < 3.31 and ≥3.31), as well as ccfDNA concentration (both continuous and dichotomized < 25.79 and ≥25.79 ng/mL). The prognostic value of combining NLR with ccfDNA concentration was also tested in the univariate and multivariate analysis, using the classification system described above. Multivariate survival analyses were performed by submitting into analysis variables that were found to be significant (*p* < 0.05) in univariate analysis and by adjusting for age and ECOG-PS, including dichotomized NLR and ccfDNA levels or combination of NLR with ccfDNA concentration.

In univariate analyses, tumor size (HR 1.02, 95% CI 1.01–1.04, *p* = 0.004); metastatic status: M0 vs. oligo-metastatic PDAC (HR 2.09, 95% CI 1.13–3.84, *p* = 0.018) and M0 vs. multi-metastatic PDAC (HR 2.53, 95% CI 1.49–4.29, *p* = 0.001); stage I/II vs. IV (HR 2.08, 95% CI 1.16–3.73, *p* = 0.013); NLR ≥ 3.31 (HR 0.58, 95% CI 0.37–0.91, *p* = 0.017); ccfDNA levels ≥ 25.79 ng/mL (HR 0.46, 95% CI 0.25–0.86, *p* = 0.015) and the combination of NLR with ccfDNA concentration: NN vs. PN (HR 1.62, 95% CI 1.01–2.62, *p* = 0.048) and NN vs. PP (HR 3.21, 95% CI 1.51–6.83, *p* = 0.002), were independent prognostic factors for OS (Table 2).

### 2.4. Higher ccfDNA Concentration and NLR Are Associated with Poorer Survival in PDAC Patients

**Figure 2:**
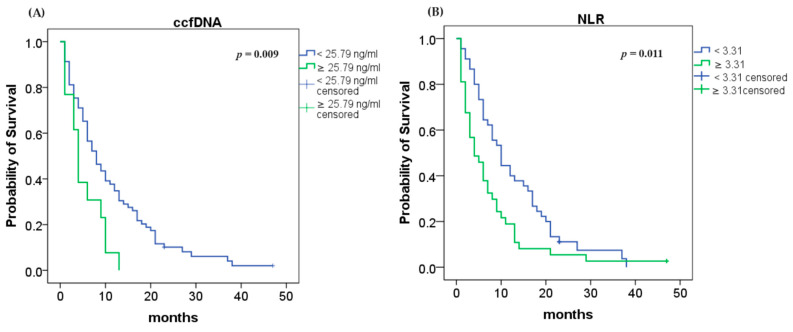
Median overall survival (OS): (**A**) OS according to ccfDNA concentration (*p* = 0.009); (**B**) OS according to NLR (*p* = 0.011).

Using the Kaplan–Meier method and the log rank test, we found that an elevated ccfDNA level (≥25.79 ng/mL) was highly associated with shorter OS (4 vs. 8 months; log rank *p* = 0.009; Figure 2A). Analyses of NLR demonstrated that higher values (≥3.31) were associated with lower OS (4 vs. 10 months; log rank *p* = 0.011; Figure 2B).

In the multivariate Cox regression model including dichotomized NLR and ccfDNA concentration, the independent prognostic factors of OS were: tumor size (HR 1.02, 95% CI 1.00–1.03, *p* = 0.049), metastatic status: M0 vs. oligo-metastatic PDAC (HR 3.78, 95% CI 1.17–12.19, *p* = 0.026) and M0 vs. multi-metastatic PDAC (HR 5.37, 95% CI 1.33–21.62, *p* = 0.018), and ccfDNA levels ≥ 25.79 ng/mL (HR 0.45, 95% CI 0.21–0.97, *p* = 0.041), as presented in Table 3.

### 2.5. The Combination of ccfDNA Concentration and NLR Values Significantly Improves Prognostic Accuracy of PDAC Patients

**Figure 3:**
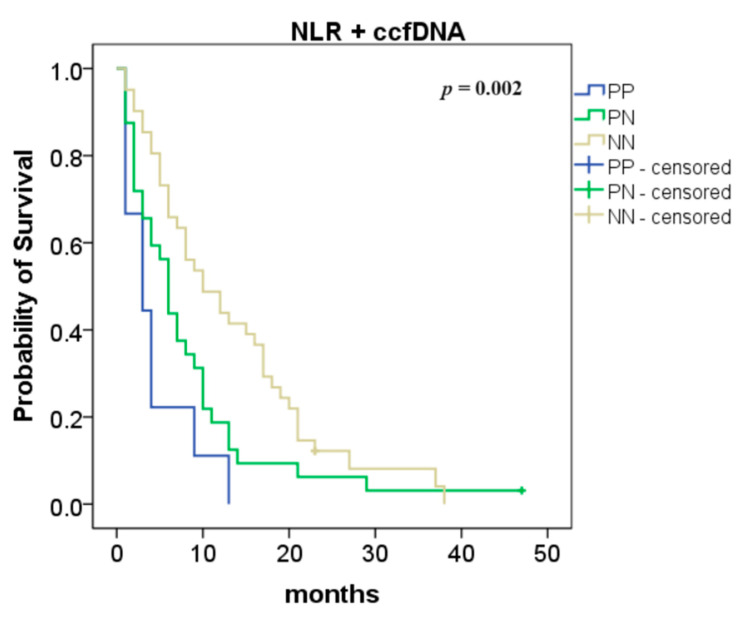
Association between neutrophil-to-lymphocyte ratio (NLR) and circulating cell-free DNA (ccfDNA) levels—overall survival according to the NLR and ccfDNA combination (both positive (PP) compared to one positive (PN): *p* = 0.038; both negative (NN) compared to both positive (PP): *p* = 0.0003).

The combination of NLR with ccfDNA levels was also analyzed and we found an improvement in the overall survival (NN vs. PP: 10 vs. 3 months, *p* = 0.0003 and NN vs. PN: 10 vs. 6 months, *p* = 0.038; Figure 3).

The multivariate analysis, including the combination of ccfDNA levels with NLR, revealed that metastatic status and the combination of ccfDNA levels with NLR were the independent prognostic factors of OS (Table 4). Metastatic (oligo- or multi-metastatic) PDAC was associated with worse OS compared to non-metastatic disease (M0 vs. oligo-metastatic PDAC: HR 3.78, 95% CI 1.17–12.21, *p* = 0.026 and M0 vs. multi-metastatic PDAC: HR 5.73, 95% CI 1.40–23.37, *p* = 0.015). Moreover, when ccfDNA levels and NLR were combined, patients with NN showed significantly longer OS than patients with PN or PP (NN vs. PN (HR 1.56, 95% CI 0.93–2.63, *p* = 0.092) and NN vs. PP (HR 2.81, 95% CI 1.11–7.15, *p* = 0.030)).

## 3. Materials and Methods

### 3.1. Study Design

This prospective observational study was carried out in a tertiary gastroenterology referral center in Bucharest, Romania, between October 2018 and December 2021. After receiving institutional review board approval (IRB: 23878/14 June 2018), 82 patients with histologically confirmed PDAC were enrolled in the study. All patients provided informed consent for blood sample collection and study enrollment.

Peripheral blood was collected from patients with pancreatic lesions who had been referred to our department for endoscopic ultrasound-guided fine needle aspiration (EUS-FNA) prior to any invasive procedure or the administration of chemotherapy. A customized form was used by two gastroenterologists to gather the clinical data, which included baseline demographics (age, gender, Eastern Cooperative Oncology Group Performance Status (ECOG-PS), presence of diabetes mellitus), tumor characteristics (size in millimeters (mm), location (head/uncinate process or body/tail), vascular invasion status (venous and/or arterial invasion), metastatic status (without metastases (M0), oligo-metastatic or multi-metastatic disease), and TNM stage according to the American Joint Committee on 8th Cancer Edition (AJCC)), as well as baseline CA 19-9 and NLR.

The diagnosis of PDAC was established by the EUS-FNA. Our criteria for classifying a PDAC as oligo-metastatic were a maximum of four metastases in either the liver or the lungs and a CA 19-9 level less than 1000 U/mL, at the time of diagnosis. The exclusion criteria for this study were the existence of a prior malignancy and a different histological diagnosis.

### 3.2. Blood Sample Collection and ccfDNA Isolation from Plasma

Twenty-five mL of peripheral blood were collected in ethylenediaminetetraacetic acid (EDTA) tubes and processed within 4 h of sampling. The DNA isolation was performed using the QIAamp^®^ MinElute^®^ ccfDNA Kit (Qiagen, Hilden, Germany) from plasma obtained after three steps of centrifugation, according to the manufacturer’s protocol. This method consists of binding the circulating DNA onto magnetic beads and further adsorbing it onto a silica membrane. A washing step was performed to ensure DNA purity. The samples were eluted in 25 μL of ultra-pure water (Qiagen) and stored at −80 °C until further analysis. The ccfDNA concentration was quantified using a Qubit 3.0 Fluorometer (Life Technologies, Kuala Lumpur, Malaysia) and measured in ng/μL, eluted in 25 μL of ultra-pure water. To calculate the ccfDNA concentration per 1 mL of plasma, we multiplied the measured concentration by the elution volume and divided the result by the plasma volume in all cases.

### 3.3. Statistical Analysis

Statistical analyses were performed using SPSS (IBM SPSS Statistics for Windows, Version 27.0 Armonk, NY, USA: IBM Corp.) and R Software version 4.0.2. All tests were two-sided, considering *p* < 0.05 for statistical significance. Missing data were automatically excluded from the analyses. Continuous variables were reported as median and interquartile range (IQR), and categorical variables as frequency (%). The Mann–Whitney U test and Pearson’s correlation coefficient were applied for categorical and continuous variables, respectively, to determine the statistical association between ccfDNA levels and tumor features, ECOG-PS, or biological parameters (CA 19-9, NLR). When more than two groups (such as tumor stage or metastatic status) were being compared, one-way analysis of variance (ANOVA) was used.

For the survival analysis, we dichotomized the concentration of ccfDNA at a cut-off of 25.79 ng/mL using a maximally selected rank statistic via conditional Monte-Carlo (R Software version 4.0.2, maxstat package) [19,20]. This allowed us to classify observations into two groups based on an ordinal predictor variable. A time-dependent receiver operating characteristic (ROC) analysis was performed using 6 month survival as the reference time point in order to determine the optimal cut-off value for baseline NLR (cut-off 3.31, AUC 0.331, *p* = 0.009). The estimation of survival rates was performed with the Kaplan–Meier method, and survival curves were compared using the log rank test, considering *p* < 0.05 for statistical significance. The multivariate survival analysis was further conducted using the Cox regression model, submitting into analysis variables that were found to be significant (*p* < 0.05) or near significant in the univariate analysis and adjusting for age and ECOG-PS. Baseline characteristics (age, gender, tumor location, size, stage, metastatic status, ECOG-PS, presence of diabetes mellitus, CA 19-9, NLR), as well as ccfDNA concentration, were analyzed with respect to their prognostic significance. The combination of NLR with ccfDNA was also analyzed and positive and negative values were assigned when NLR or ccfDNA were above (positive) or below (negative) the cut-off for prognostic value in OS; we defined three groups: both positive (PP), one positive (PN), both negative (NN) [21]. Overall, survival was defined as the time from diagnosis to death due to pancreatic cancer-related complications or the end of follow-up. Survival data were censored at the last follow-up. The cut-off date for follow-up was 15 May 2023.

## 4. Discussion

While the clinical utility of ccfDNA concentration as a screening tool is low, studies on pancreatic, prostate, breast, and colorectal cancer have demonstrated that total ccfDNA concentration is a cost-effective technique for prognosis, monitoring during treatment, and monitoring for recurrence [12,17,22]. Assessing tumor burden with circulating tumor cells (CTCs) and circulating tumor DNA (ctDNA) is frequently more accurate. Clinical trials have demonstrated a high specificity for detecting cancer, which makes the use of ctDNA detection for other aspects of clinical management, such as screening and minimal residual disease, attractive. However, low sensitivity is a result of a number of variables, including low tumor burden and sampling bias [23,24,25].

Plasma ccfDNA has proven to be a reliable predictor of mortality [26] as well as a biomarker that can reveal details about a variety of health disorders [27] and aging-related illnesses. A study by Jylhävä concluded that the age-related increase in cellular senescence and death rate was manifested as elevated plasma ccfDNA and higher concentrations of total ccfDNA and unmethylated ccfDNA were directly associated with inflammation, indicating that the plasma levels of these ccfDNA species were higher in older people [27]. Moreover, elevated ccfDNA plasma concentrations have been reported to be associated with vascular invasions and tumor burden in various types of malignancies. [28,29,30,31].

In a prospective cohort study including 74 patients with advanced or metastatic pancreatic cancer, high levels of total ccfDNA were associated with new distant metastasis (91% sensitivity, 95% specificity). Additionally, the researchers discovered a negative correlation between total ccfDNA concentration and OS and progression-free survival (PFS) [32].

In this study, the relationship of ccfDNA levels to age, sex, tumor features, ECOG-PS, tumor stage, metastatic status, or biological parameters (CA 19-9, NLR) was assessed. Our findings indicated a correlation between elevated plasma ccfDNA levels and age over 55 years, venous and arterial invasion, and increased number of metastases.

Neutrophil extracellular traps (NET) have been the subject of numerous recent studies that have examined their function in angiogenesis, metastasis, and tumor formation [33,34]. The mechanism known as NETosis, which occurs when activated neutrophils release chromatin, may reveal unanticipated roles for neutrophils in the development of cancer and offer an additional explanation for the higher release of ccfDNA in the bloodstream in pathologic settings. Some authors speculated that the tumor’s inflammatory/hypoxic environment could lead to neutrophil recruitment, which would raise the overall concentration of ccfDNA by causing NETs to deposit on the microvasculature and trap cancer cells in the bloodstream, as well as by NETosis [35]. There is growing evidence that tumors can modify neutrophils, even at an early stage of differentiation, to produce various functional and phenotypic polarization states that can change the behavior of the tumor [36].

The NLR had prognostic value for a variety of tumor types, and elevated baseline NLR was associated with poor OS [9,37]. In several studies, the multivariate analysis showed that patients with elevated NLR (cut-off values ranging from 2 to 5) had a worse prognosis than those with lower NLR [38,39].

Some authors have also highlighted that elevated ccfDNA levels and high NLR were related to a poor prognosis in advanced pancreatic cancer [15,38,40,41,42,43].

We previously reported in a retrospective study, which included 83 metastatic PDAC patients, that higher levels of NLR were significantly associated with shorter OS and PFS [43]. Since overfitting the cut-off values to test datasets is a well-recognized issue in statistical analyses, especially when dealing with prognostic factors, we have further used this independent cohort to validate the NLR cut-off value identified in the present study. Consequently, we could demonstrate that a lower OS (8 vs. 11 months; log rank *p* = 0.047) was linked to a NLR ≥ 3.31. The multivariate survival analysis was further conducted using the Cox regression model, including in the analysis CA 19-9 levels (≥39 U/L vs. <39 U/L), tumor location and NLR. Once more, baseline NLR ≥ 3.31 (HR 0.605, 95% CI 0.372–0.985, *p* = 0.043) was an independent predictor of OS.

In this study, the cut-off value of ccfDNA concentration was 25.79 ng/mL, which is comparable to those used in published papers. For example, in a recent study, including 61 metastatic PDAC patients, higher ccfDNA levels (>26.46 ng/mL) were associated with poorer OS and shorter PFS [44]. Using the same cut-off for ccfDNA levels, the authors replicated their findings the following year with a different cohort of 58 PDAC patients, and they observed differences in OS (172 versus 339 days; *p* = 0.0169) depending on ccfDNA concentration [21]. Since we did not have the possibility to externally validate the cut-off value of 25.79 ng/mL on another cohort of patients, we have used bootstrapping to internally validate this cut-off, using 1000 bootstrap samples. The analysis indicated that our cut-off value is robust, with a two tailed *p*-value for the regression coefficient in the Cox regression analysis of 0.002. Similarly, internal validation was positive also for NLR, with a significant *p*-value of 0.03.

A previous study demonstrated a relation between NLR and ccfDNA concentration in metastatic PDAC and showed that the combination of NLR with plasma ccfDNA levels greatly improves prognostic power and provides accurate survival risk stratification [21]. In agreement with other reports, our study supports the value of ccfDNA levels and NLR as prognostic factors in PDAC. Therefore, our results reveal that a higher ccfDNA concentration in plasma (>25.79 ng/mL) predicts a poor prognosis; similarly, patients with a high baseline NLR (>3.31) had a shorter OS compared to patients with a low NLR.

To see if it provided additional prognostic value, we performed survival analysis on the combination of baseline ccfDNA levels and NLR. The survival analyses demonstrated that OS was significantly prolonged in patients negative for both markers, compared to patients that were positive for one or two markers.

Both univariable and multivariable Cox regression analyses were performed to estimate the prognostic role of ccfDNA levels and NLR on survival, relative to other clinicopatological parameters. The univariable regression analyses confirmed the prognostic impact of ccfDNA levels and NLR, as demonstrated by the log rank test. In addition, the univariable analysis showed that tumor size, metastatic status, and tumor stage also had prognostic value.

The multivariable Cox regression analyses demonstrated that the dichotomized ccfDNA levels was an independent prognostic factor for OS. The tumor size and the absence of metastases at the time of diagnosis were also identified as prognostic factors for OS. Moreover, the multivariate analysis demonstrates that the combination of ccfDNA levels with NLR greatly helped in the prognostic stratification of PDAC patients. No other factors were identified as significant predictors of survival in the multivariable analysis.

Previous studies assessing the prognostic value of plasma ccfDNA levels included cohorts of patients who either had resectable disease or had locally advanced and/or metastatic PDAC. Patients with PDAC in all stages (resectable, borderline, locally advanced, and metastatic) were included in this study. We were able to demonstrate that the cut-off values of the DNA concentration and NLR are independent predictors of survival regardless of disease stage, despite the fact that the study was conducted in a single center with a small number of patients.

We showed that, independent of tumor stage, the quantification of ccfDNA levels provides a non-invasive, straightforward technique for predicting clinical prognosis in patients with PDAC.

To our knowledge, this is the first study in Romanian population to assess the prognostic role of the ccfDNA concentration in PDAC patients. Moreover, this is one of the first studies to demonstrate the predictive value of systemic inflammation in combination to ccfDNA concentration. Future research is required to further explore the therapeutic applicability of these findings.

While this study successfully demonstrated that increased plasma ccfDNA concentration and NLR were correlated with decreased survival, this research has potential limitations as it is a single-center study with a relatively small number of patients. The heterogeneity of the analyzed group comprising patients in different disease stages should be considered a strength of our study as it facilitated the prognostic assessment of ccfDNA and NLR as independent prognostic indicators along to already validated traditional prognostic indicators as the disease stage.

## 5. Conclusions

In summary, our study supports ccfDNA concentration and NLR as promising tools for the non-invasive prognostic assessment of PDAC patients and demonstrates that the combination of NLR with ccfDNA levels significantly improves prognostic ability and provides accurate survival risk stratification. Subsequent studies should validate this combination as a prognostic indicator in PDAC patients and assess its utility for guiding therapeutic decisions.

## Figures and Tables

**Table 1:** Baseline characteristics of the patients with PDAC.

Variable	All Patients (*n* = 82)
Median age	67 (62–70)
Age ≥ 55 years, *n* (%)	73 (89.02)
Sex, male, *n* (%)	45 (54.88)
ECOG-PS, *n* (%)	
0/1	76 (92.7)
2/3	6 (7.3)
CA 19-9, *n* (%)	
<39 U/mL	24 (29.27)
≥39 U/mL	55 (67.07)
Missing data	3 (3.66)
Median tumor size (mm)	40 (32.75–50)
Primary tumor location, *n* (%)	
Head/uncinate process	51 (62.2)
Body/tail	31 (37.8)
Vascular involvement, *n* (%)	
Venous invasion	56 (68.29)
Arterial invasion	36 (43.9)
Tumor stage *, *n* (%)	
IA	1 (1.2)
IB	8 (9.8)
IIA	2 (2.4)
IIB	8 (9.8)
III	21 (25.6)
IV	42 (51.2)
Metastatic status, *n* (%)	
No metastases	35 (42.7)
Oligo-metastatic	17 (20.7)
Multi-metastatic	30 (36.6)
ccfDNA concentration (ng/mL)	10.3 (5.1–21.46)
NLR	3.22 (2.34–4.42)

Annotations: all variables are provided as the median (IQR) or number (percentage); * according to AJCC 8th edition [18]. Abbreviations: ECOG-PS: Eastern Cooperative Oncology Group Performance Status, CA 19-9: carbohydrate antigen 19-9, ccfDNA: circulating cell-free DNA, NLR: neutrophil-to-lymphocyte ratio.

**Table 2:** Univariate Cox Regression Analysis.

Prognostic Factor	*p*	HR (95% CI)
Age (years)	0.446	0.99 (0.96–1.02)
Age > 55 years	0.084	1.88 (0.92–3.85)
Sex: Male vs. Female	0.242	0.76 (0.48–1.20)
Diabetes	0.415	0.82 (0.51–1.32)
Baseline ECOG-PS (0/1 vs. 2)	0.057	2.28 (0.98–5.34)
Tumor size	0.004	1.02 (1.01–1.04)
Tumor location:		
Head vs. Body	0.623	0.88 (0.53–1.46)
Head vs. Tail	0.19	1.66 (0.78–3.53)
Metastatic status:		
M0 vs. Oligo-metastatic	0.018	2.09 (1.13–3.84)
M0 vs. Multi-metastatic	0.001	2.53 (1.49–4.29)
Tumor stage:		
Stage I/II vs. Stage III	0.717	1.13 (0.59–2.16)
Stage I/II vs. Stage IV	0.013	2.08 (1.16–3.73)
CA 19-9 ≥ 39 UI/mL vs. <39 UI/mL	0.377	0.79 (0.49–1.31)
NLR ≥ 3.31 vs. NLR < 3.31	0.017	0.58 (0.37–0.91)
ccfDNA concentration (continuous)	0.104	1.01 (0.99–1.03)
ccfDNA levels ≥ 25.79 ng/mL vs. <25.79 ng/mL	0.015	0.46 (0.25–0.86)
Combination of ccfDNA levels with NLR		
NN vs. PN	0.048	1.62 (1.01–2.62)
NN vs. PP	0.002	3.21 (1.51–6.83)

Abbreviations: ECOG-PS: Eastern Cooperative Oncology Group Performance Status, CA 19-9: carbohydrate antigen 19-9, M0: no metastases, ccfDNA: circulating cell-free DNA, NLR: neutrophil-to-lymphocyte ratio, NN: both biomarkers negative, PN: one biomarker positive, PP: both biomarkers positive.

**Table 3:** Multivariate Cox Regression Analysis (including dichotomized NLR and plasma ccfDNA concentration).

Prognostic Factor	*p*	HR (95% CI)
Age > 55 years	0.27	1.56 (0.71–3.45)
Baseline ECOG-PS (0/1 vs. 2)	0.748	0.85 (0.32–2.29)
Tumor size	0.049	1.02 (1.00–1.03)
Metastatic status:		
M0 vs. Oligo-metastatic	0.026	3.78 (1.17–12.19)
M0 vs. Multi-metastatic	0.018	5.37 (1.33–21.62)
Tumor stage:		
Stage I/II vs. Stage III	0.6	0.83 (0.41–1.69)
Stage I/II vs. Stage IV	0.136	0.35 (0.09–1.39)
NLR ≥ 3.31 vs. NLR < 3.31	0.192	0.71 (0.43–1.19)
ccfDNA levels ≥ 25.79 ng/mL vs. <25.79 ng/mL	0.041	0.45 (0.21–0.97)

Abbreviations: ECOG-PS: Eastern Cooperative Oncology Group Performance Status, M0: no metastases, NLR: neutrophil-to-lymphocyte ratio, ccfDNA: circulating cell-free DNA.

**Table 4:** Multivariate Cox Regression Analysis (including combination of NLR with ccfDNA concentration).

Prognostic Factor	*p*	HR (95% CI)
Age > 55 years	0.364	1.44 (0.66–3.17)
Baseline ECOG-PS (0/1 vs. 2)	0.894	0.93 (0.34–2.56)
Tumor size	0.057	1.02 (1.00–1.03)
Metastatic status:		
M0 vs. Oligo-metastatic	0.026	3.78 (1.17–12.21)
M0 vs. Multi-metastatic	0.015	5.73 (1.40–23.37)
Tumor stage:		
Stage I/II vs. Stage III	0.561	0.81 (0.39–1.65)
Stage I/II vs. Stage IV	0.104	0.32 (0.08–1.26)
Combination of ccfDNA with NLR		
NN vs. PN	0.092	1.56 (0.93–2.63)
NN vs. PP	0.030	2.81 (1.11–7.15)

Abbreviations: ECOG-PS: Eastern Cooperative Oncology Group Performance Status, M0: no metastases, ccfDNA: circulating cell-free DNA, NLR: neutrophil-to-lymphocyte ratio, NN: both biomarkers negative, PN: one biomarker positive, PP: both biomarkers positive.

## Data Availability

Data are available upon reasonable request and with the permission of the corresponding author.
